# Description of physical activity and screen time among U.S. middle school students

**DOI:** 10.1371/journal.pone.0311103

**Published:** 2025-02-19

**Authors:** Mi Zhou, Zikun Zhao, Xiaoran Li, Yanxuan Jin, Xinlei Hong, Haoning He, Mengchu Zhao, Xiaomei Song

**Affiliations:** 1 Department of Health and Physical Education, The Education University of Hong Kong, Hong Kong, China; 2 Faculty of Physical Education and Sport, Beijing Normal University, Beijing, China; 3 Wushu School, Beijing Sports University, Beijing, China; 4 Faculty of Educational Studies, Universiti Putra Malaysia, Putrajaya, Serdang, Malaysia; 5 School of Sport, Exercise and Health Sciences, Loughborough University, Loughborough, United Kingdom; 6 Department of Rehabilitation Medicine, Shanghai Sixth People’s Hospital Affiliated to Shanghai Jiaotong University School of Medicine, Shanghai, China; 7 Department of Nursing, The Second Affiliated Hospital of Soochow University, Suzhou, Jiangsu, China; Universidad Santiago de Cali, COLOMBIA

## Abstract

**Background:**

Physical activity (PA) is important for students in secondary school, however, trends in PA among secondary school students have shown a significant decline. There is a need to understand the PA of middle school students.

**Objective:**

The first objective is to identify the PA levels and screen time of students in middle school. The second objective of the study is to examine the PA levels and screen time among students of different genders.

**Methods:**

Participants from four consecutive two-year cycles of National Health and Nutrition Examination Survey (NHANES, 2011–2012, 2013–2014, 2015–2016, and 2017–2018) were included in this study. Spearman correlation model was used to identify the correlation between participants’ demographics, PA, and screen time data. Negative binomial regression model was used to describe students’ PA and screen time (Dependent variable) in different grades (Independent variables). Gender and Age were taken as control variables.

**Results:**

After the data preprocessing, 2516 participants were included in this study. A significant correlation has been found between grade and PA, instead of screen time. Negative binomial regression shows that students have the lowest PA in their transition year grade 6, and their screen time decreased with the grade increased. Significant differences can be found across gender. Future efforts should focus on developing school transition support programs designed to improve PA.

## Introduction

Physical activity (PA) is defined as any bodily movement produced by skeletal muscles that results in energy expenditure [1]. For students, engaging in PA both within and outside the classroom not only promotes overall health but also boosts motivation, sparks interest in learning, and enhances academic performance [2]. Recent research indicates that PA helps students regulate body weight, reduce blood pressure [3], enhance bone health [4], and increase muscle strength and functionality [5]. Furthermore, it contributes to alleviating anxiety and depression, thereby improving emotional stability and mental resilience [6].

However, global trends indicate a significant decline in PA among adolescents [7]. A majority of adolescents fail to meet existing guidelines for PA [8]. In 27 countries, the prevalence of insufficient PA exceeds 90% [7]. Adolescents aged 11–15, aligning with the middle school age group, exhibit the highest levels of insufficient PA. Worldwide, approximately 81.0% (95% CI: 77.8–87.7) of middle school students do not engage in adequate PA, with 77.6% of boys (76.1–80.4) and 84.7% of girls (83.0–88.2) falling below the recommended levels. In the United States, the PA levels among middle school students saw a decline from 2011 to 2017 [9]. Given that middle school students are in a pivotal phase of physical development, sufficient PA is crucial for the healthy development of bones, muscles, and cardiovascular functions. Moreover, the middle school years are vital for fostering healthy lifestyle habits and establishing enduring PA routines [10]. Thus, there is a pressing need to comprehend and support the PA of middle school students.

Numerous studies have indicated that students’ PA decreases as they age [11]. However, there is a paucity of research on differences in PA between grades in middle school. Considering that middle school is a critical period of transition from childhood to adolescence, during which students’ behaviours may change significantly in a short period of time [12, 13], it is essential to examine PA differences between grades at this stage.

The purpose of this study was to explore the significant differences between students at different grade levels and to investigate the PA levels of students at different grade levels in the United States, providing a comprehensive understanding of the variations in these levels. The goal of this study is twofold. The first goal is to identify and compare the PA levels of students among students in grade 5 through 9, highlighting any grade-specific trends or patterns in PA. Given that some research suggests that screen time is an important factor in PA and that changes in PA may vary by gender, the second goal of the study is to examine the PA levels and screen time among students of different genders within the same grade range. The study will provide insights into gender-related differences in PA during these formative school years. The findings of the study will help enrich the existing body of knowledge about student PA and may inform the future development of educational and health policies that promote PA in school-aged children.

## Research design

We used a cross-sectional study design and utilized data from the National Health and Nutrition Examination Survey (NHANES). NHANES has been a major program of the National Center for Health Statistics (NCHS) since the early 1960s. NHANES collects data on hundreds of variables to determine the prevalence of major diseases and risk factors aimed at promoting health and preventing disease [14]. NHANES surveys thousands of non-institutionalized civilians across the country to provide a nationally representative sample. The collected information is posted online in the form of data files and used in a variety of epidemiologic studies. The findings of the study contribute to the formulation of public health policies and programs [14].

Data from four consecutive two-year cycles were used in this study (NHANES 2011–2012, 2013–2014, 2015–2016, and 2017–2018). Prior to the 2011–2012 cycle, NHANES changed the PA measure. Data collection after the 2017–2018 cycle was incomplete due to the impact of the epidemic and the fact that the epidemic would have significantly impacted PA data, so it was not included in this study. Therefore, additional years could not be included in this study. Written consent was provided by each participant. The NCHS Ethics Review Board approved the measurement procedures and data collection and authorized the online release of the data for public use.

The NHANES subjects in this study were U.S. students in grades 5 through 9. completely answered the three questions in the PA section were included in the analysis: PAQ715: "Number of hours of computer use in the past 30 days," PAQ710: "Number of hours of television or video viewing in the past 30 days," and PAQ706: "Number of days with at least 60 minutes of physical activity" or students who answered the PA-Youth section completely. They also needed complete data on gender, age, and grade level. A total of 2516 participants provided complete data for each variable and were included in the analysis.

## Data analysis

Descriptive data are presented as mean ± standard error (M ± SE) for continuous variables and percentages ± SE for categorical variables. Gender, age, and grade level of the included sample were reported. In this study, PAQ706, PAQ710, and PAQ715 were the dependent variables, grade level was the categorical independent variable, and gender and age were the control variables. Since PAQ706, PAQ710, and PAQ715 were discrete data, they were analyzed using Poisson regression. If the model exhibited excessive dispersion, the data were analyzed using negative binomial regression to examine the effect of grade level on PA. To investigate the differences in PA by gender across grades. A gender*grade interaction effect is also reported. All regression results were presented as regression coefficients, SE, test statistics, and p-values. R language will be used to conduct the analysis. The code for analyzing the dataset can be found in the Open Science Framework (https://osf.io/z72qa/).

## Results

### Description of demographics and PA results

Table 1 demonstrates the demographic and PA results of the participants. There was a total of 2,516 participants in this study with an equal distribution of males and females (50% each). Participants were distributed across the five grade levels as follows: Year 5 (29.5%), Year 6 (19.1%), Year 7(19.2%), Year 8 (19.3%), and Year 9(13.0%).

**Table 1:** Description of demographics and PA results.

Parameters	Levels	Stats
Gender	Male	1257 (50.0%)
	Female	1259 (50.0%)
Grade	Year5	742 (29.5%)
	Year6	480 (19.1%)
	Year7	482 (19.2%)
	Year8	485 (19.3%)
	Year9	327 (13.0%)
Age	Mean ± SD	12.8 ± 1.6
PAQ706	Mean ± SD	4.2 ± 2.3
PAQ710	Mean ± SD	2.5 ± 1.8
PAQ715	Mean ± SD	2.8 ± 2.5

The correlation among demographic variables (Gender, Age, Grade), physical activity (PAQ706), and screen time (PAQ710 and PAQ715) can be found in Table 2.

**Table 2:** Correlation among demographics, PA, and screen time.

	Gender	Age	Grade	PAQ706	PAQ710	PAQ715
**Gender**						
**Age**	-0.004					
**Grade**	-0.058**	0.028				
**PAQ706**	-0.116***	0.040*	-0.175***			
**PAQ710**	0.007	0.036	0.017	-0.105***		
**PAQ715**	0.008	-0.001	0.109***	-0.102***	0.235***	

Computed correlation used spearman-method with listwise-deletion. The significance codes are as follows

*** p < 0.001

** p < 0.01

* p < 0.05.

### PA and screen time in each grade

As shown in Table 3, there was a significant decline in the number of days that grade 6 were active for at least 60 minutes per week (b = -0.0728, z = -2.00, p = 0.045), with an OR of 0.930, indicating that students grade 6 were approximately 7% less likely to be active for at least 60 minutes per week than grade 5. PA increased as grade level increased, with a significant increase in the number of days in grade 9 (b = 0.1983, z = 2.62, p = 0.009), with an OR of 1.219, indicating that students grade 9 were approximately 22% more likely to be active for at least 60 minutes per week than grade 5.

**Table 3:** Negative binomial regression results of days physically active at least 60 min (PAQ706).

Predictor	Coefficients	Std..Error	z.value	Odds.Ratio	P.value
(Intercept)	2.813	0.188	14.971	16.660	0.000***
YEAR6	-0.073	0.036	-2.003	0.930	0.045*
YEAR7	0.032	0.049	0.669	1.033	0.504
YEAR8	0.099	0.061	1.616	1.104	0.106
YEAR9	0.198	0.076	2.621	1.219	0.009**

Note: The significance codes are as follows

*** p < 0.001

** p < 0.01

* p < 0.05.

As shown in Tables 4 and 5, there was a decrease in screen time in grade 6 and a significant decrease in grade 7 compared to grade 5 (PAQ710: b = -0.170, z = -2.623, p = 0.009; PAQ715: b = -0.162, z = -2.012, p = 0.044). Grade 8 spent more time watching TV than grade 6 and grade 7, while grade 9 spent less time in this area (b = -0.179, z = -1.805, p = 0.071). However, they spent more on computers in grade 9 (b = -0.125, z = -1.007, p = 0.314).

**Table 4:** Results of negative binomial regression on hours watch TV or videos past 30 days (PAQ710).

Predictor	Coefficients	Std..Error	z.value	Odds.Ratio	P.value
(Intercept)	0.230	0.248	0.930	1.259	0.352
YEAR6	-0.064	0.049	-1.309	0.938	0.191
YEAR7	-0.170	0.065	-2.623	0.844	0.009**
YEAR8	-0.096	0.080	-1.200	0.908	0.230
YEAR9	-0.179	0.099	-1.805	0.836	0.071

Note: The significance codes are as follows

*** p < 0.001

** p < 0.01

* p < 0.05.

**Table 5:** Results of negative binomial regression on hours use computer past 30 days (PAQ715).

Predictor	Coefficients	Std.Error	z.value	Odds.Ratio	P.value
(Intercept)	-0.164	0.312	-0.528	0.849	0.598
YEAR6	-0.121	0.062	-1.951	0.886	0.051
YEAR7	-0.163	0.081	-2.012	0.850	0.044*
YEAR8	-0.140	0.101	-1.384	0.869	0.166
YEAR9	-0.125	0.124	-1.007	0.882	0.314

Note: The significance codes are as follows: *** p < 0.001, ** p < 0.01, * p < 0.05.

### Gender differences in PA and screen time across grades

As shown in Table 6, boys’ PA levels decreased slightly compared to grade 5(b = -0.079, z = -1.623, p = 0.104), but continued to increase from grade 7 to grade 9(grade 7: b = 0.081, z = 1.416, p = 0.157; grade 8: b = 0.202, z = 2.907, p = 0.004). In contrast, girls’ PA levels generally declined with age, especially in grade 8 (b = -0.093, z = -1.453, p = 0.146) and grade 9 (b = -0.206, z = -3.180, p = 0.001), which was significant compared to grade 5.

**Table 6:** Negative binomial regression results on gender difference in days physically active at least 60 min (PAQ706).

Predictor	Coefficients	Std.Error	z.value	Odds.Ratio	P.value
(Intercept)	2.786	0.187	14.878	16.216	0.000***
YEAR6	-0.079	0.049	-1.623	0.924	0.104
YEAR7	0.081	0.057	1.416	1.084	0.157
YEAR8	0.202	0.070	2.907	1.224	0.004**
YEAR9	0.278	0.082	3.371	1.320	0.001**
GENDER	-0.080	0.038	-2.106	0.923	0.035*
YEAR6:GENDER	0.023	0.064	0.358	1.023	0.720
YEAR7:GENDER	-0.093	0.064	-1.453	0.911	0.146
YEAR8:GENDER	-0.206	0.065	-3.180	0.814	0.001**
YEAR9:GENDER	-0.169	0.075	-2.272	0.845	0.023*

Note: The significance codes are as follows

*** p < 0.001

** p < 0.01

* p < 0.05.

As shown in Tables 7 and 8, boys’ screen time decreased slightly in grade 6 compared to grade 5 (PAQ710: b = -0.085, z = -1.283, p = 0.200; PAQ715: b = -0.184, z = -2.165, p = 0.677), but gradually increased from grade 7 (b = -0.201, z = -2.545, p = 0.011) to grade 9 (b = -0.151, z = -1.375, p = 0.169). In contrast, girls’ screen time generally decreased with age, especially in grade 8 and grade 9.

**Table 7:** Negative binomial regression results on gender difference in hours watch TV or videos past 30 days (PAQ710).

Predictor	Coefficients	Std.Error	z.value	Odds.Ratio	P.value
(Intercept)	0.235	0.248	0.948	1.265	0.343
YEAR6	-0.085	0.067	-1.283	0.919	0.200
YEAR7	-0.201	0.079	-2.545	0.818	0.011*
YEAR8	-0.211	0.094	-2.246	0.810	0.025*
YEAR9	-0.151	0.110	-1.375	0.860	0.169
GENDER	0.000	0.054	0.009	1.000	0.993
YEAR6:GENDER	0.035	0.086	0.403	1.036	0.687
YEAR7:GENDER	0.049	0.087	0.566	1.050	0.571
YEAR8:GENDER	0.201	0.085	2.378	1.223	0.017*
YEAR9:GENDER	-0.094	0.097	-0.962	0.910	0.336

Note: The significance codes are as follows

*** p < 0.001

** p < 0.01

* p < 0.05.

**Table 8:** Negative binomial regression results on gender difference in hours watch TV or videos past 30 days (PAQ715).

Predictor	Coefficients	Std..Error	z.value	Odds.Ratio	P.value
(Intercept)	-0.130	0.312	-0.417	0.878	0.677
YEAR6	-0.184	0.085	-2.165	0.832	0.030*
YEAR7	-0.250	0.099	-2.527	0.779	0.012*
YEAR8	-0.246	0.118	-2.087	0.782	0.037*
YEAR9	-0.213	0.138	-1.542	0.808	0.123
GENDER	-0.011	0.069	-0.157	0.989	0.875
YEAR6:GENDER	0.111	0.110	1.012	1.117	0.312
YEAR7:GENDER	0.156	0.109	1.437	1.169	0.151
YEAR8:GENDER	0.184	0.107	1.720	1.202	0.085
YEAR9:GENDER	0.153	0.120	1.275	1.165	0.202

Note: The significance codes are as follows

*** p < 0.001

** p < 0.01

* p < 0.05.

## Discussion

The first goal of this study is to identify and compare the PA and screen time among grade 5–9. The results indicate that PA is at its lowest in grade 6, the middle school transition year. We believe this may be due to the effects of school transition. School transitions can be defined as the process of moving from one educational institution to another and is considered important and challenging for students [15, 16]. In the United States, this transition usually occurs between grade 5 and grade 6, at approximately 11 to 12 years of age. During this period, students must adjust to a new school environment, different academic expectations, and new social dynamics, making it one of the most stressful events in a young person’s life [17]. Previous research indicated that academic pressure and the subsequent reduction in leisure time negatively impact children’s PA levels, leading to an increase in sedentary lifestyles and screen time, which is associated with poorer physical health outcomes [18–20].

Contrary to prevailing research suggesting a decline in PA with advancing age [21, 22], this study noted an increase in PA levels in grades 7, 8, and 9. This anomaly may stem from variations in the types of physical activities made accessible to students at different educational stages. Specifically, sixth-grade students are generally navigating the transition from childhood to adolescence, a period during which physical education programs often focus on enhancing physical fitness and mastering fundamental motor skills [23–25]. As a result, the curriculum and the scope of physical activities in the early grades are relatively limited [26]. However, as students progress to higher grades, the educational focus transitions towards the application of complex motor skills and the development of strategic competencies in sports [27]. This advancement is marked by an increased participation in sophisticated team sports such as basketball, soccer, and volleyball. Additionally, students dedicate more time to extracurricular activities that require team collaboration and tactical training, significantly enhancing their levels of PA. As physical maturation and athletic proficiency among students improve, opportunities for participation in intramural, interscholastic, and regional sports competitions expand [28]. Success in these competitive environments necessitates increased commitment to post-school training sessions, which, in turn, further augments their PA levels, thus creating a positive feedback loop.

Since the decline in PA occurs only in the first year of transition, future efforts should focus on developing school transition support programs designed to improve PA. These programs will help students through the most challenging part of the transition and prepare them for the rest of middle and high school. However, the majority of current school transition support programs focus on students’ social and emotional health, with little focus on PA. Future research should develop more PA-supportive school transition programs. For students in grades 7–9, the consistent increase in PA levels suggests a reduced cause for concern. However, mean values can mask underlying disparities. Significant increases in PA among certain students may obscure the exclusion of others from team sports, leading to insufficient PA. Future research should aim to identify the characteristics of these excluded students and develop targeted interventions to enhance their PA levels.

Previous research also indicates that the reduction in PA may be related to an increase in screen time. The widespread use of electronic devices has led to more sedentary activities [29]. Activities such as watching TV, using computers, and playing mobile games are gradually replacing traditional outdoor games and sports [30]. A study on PA and screen time has documented this shift, highlighting a clear correlation between increased screen use and decreased PA [31]. Interestingly, our study found no increase in screen time, including television viewing and computer use; in fact, screen time decreased significantly, reaching a nadir in grade 7 before rising again in grade 8. This inconsistency suggests that the changes in screen time do not reflect changes in PA. Given that students are neither increasing their PA nor their screen time, it raises the question of where their time is being spent. We argue that changes in screen time are influenced not only by academic stress but also by changes in social relationships. Transitioning to a new and unfamiliar environment requires students to invest time in building new friendships. Previous research has shown that peer influences and social activities have a significant impact on adolescents’ screen time, with students preferring non-screen social activities [32]. Consequently, screen entertainment time decreases. By grade 8, students are fully acclimated to their new environments and social circles, allowing for more time for screen activities. However, by grade 9, the increased academic pressure on students about to transition from middle school to high school leads to another decrease in screen recreation time [33]. Based on this finding, the decline in students’ PA cannot be attributed simply to increased sedentary and screen time. In addition, students’ PA and screen time are likely to be influenced by the school environment (academic and social aspect), which further justifies the need for PA intervention programs in schools. However, due to the limitations of the experimental design, we were unable to consider the potential impact of the interaction between school and adolescence on students’ PA. Future research needs to examine PA across grades and ages.

Supriyanto found that male students were more inclined to participate in sports and competitions during their leisure time, whereas female students tend to participate in low-intensity or social activities, resulting a gender disparity in PA [34]. This study reached similar conclusions, indicating that male students’ PA showed a gradual increase with age, while female students’ PA decreased with age. We believe that the possible reason for this result is that female students usually face more time management challenges, possibly due to greater responsibilities in family and social roles, reducing the time they can allocate to PA [35]. Additionally, female students often tend to be more concerned with body image, and their concern for appearance and self-esteem can affect their participation in physical activities and their concern for appearance and self-esteem can affect their participation in physical activities. In contrast, male students are more concerned with physical fitness and competitiveness [36].

It is generally accepted that males typically use video games more frequently as a means of social interaction [37], and growing up they tended to choose video games and PA as their primary forms of entertainment [38]. This resulted in boys having more screen time than girls. However, the results of this study differ from previous findings. Compared to grade 5, boys’ screen time decreased sharply in grade 6, reaches its lowest point in grade 7, and then slightly increases from grade 8 to grade 9. Conversely, screen time for girls continued to rise as they entered middle school and did not decrease until grade 9.

In addition to the effects of sociocultural expectations mentioned earlier, these results may indicate that screen time becomes more significant for female students during middle school. Studies have found that female students are more inclined to use social media to communicate and build social relationships [39], and social media is a crucialmean for them to fit into groups [40]. The increase in screen time for girls during middle school may be related to their desire to enhance social interactions and integrate into peer groups.

Another interesting aspect in the results is that decreased PA and screen time for boys may indicate that PA and screen time are not strongly correlated for middle school boys, who tend to spend time on other activities. However, a significant correlation between PA and screen time was found for girls. A decrease in PA led to an increase in screen time. This suggests that if the goal is to reduce girls’ screen time, increasing their PA levels is the best option. In summary, bearing in mind the poor performance of female students in both PA and screen time, more targeted interventions need to be designed to help girls increase their PA and reduce their screen time.

Some limitations are associated with our findings. To begin with, this study was a cross-sectional study that identified only PA and screen time for students of different ages. While we compared their results to some extent, this does not determine the true trend of students’ PA as they age. This needs to be further verified through longitudinal studies in the future. Second the sample size of this study is relatively limited. Despite the relatively balanced gender ratio and age, the total sample size is only 2000. A larger sample size is needed for future studies to confirm our findings. To conclude, the screen time and PA measures used in this study were obtained through questionnaires, not PA accelerometers. Such self-reported data may be subject to recall bias and may not accurately represent students’ PA and screen time.

## Conclusion

This study explored trends in PA and screen time among U.S. middle school students across grades 5 through 9. The findings revealed a significant decline in PA levels during the transition year (grade 6), which was followed by a gradual increase in subsequent years. Conversely, screen time exhibited variability across grades, with pronounced gender differences; girls engaged in screen-based activities more frequently than boys. These observations underscore the necessity for school transition programs that prioritize physical health. It is imperative to devise targeted interventions for vulnerable demographics, particularly girls and students less inclined to participate in team sports. Future research should adopt a longitudinal approach and incorporate larger cohorts to further investigate these trends and formulate effective strategies to foster healthy behaviors among students.

## Supporting information

S1 Code R code for data analysis. (R)
